# High biodiversity in a limited mountain area revealed in the traditional production of Historic Rebel cheese by an integrated microbiota–lipidomic approach

**DOI:** 10.1038/s41598-021-89959-x

**Published:** 2021-05-14

**Authors:** Federica Turri, Paola Cremonesi, Giovanna Battelli, Marco Severgnini, Milena Brasca, Gustavo Gandini, Flavia Pizzi

**Affiliations:** 1 Institute of Agricultural Biology and Biotechnology, National Research Council, via Einstein, 26900 Lodi, Italy; 2 Institute of Sciences of Food Production, National Research Council, via Giovanni Celoria 2, 20133 Milan, Italy; 3 Institute of Biomedical Technologies, National Research Council, via Fratelli Cervi 93, 20090 Segrate, Italy; 4 Department of Veterinary Medicine, University of Milan, via dell’Università 6, 26900 Lodi, Italy

**Keywords:** High-throughput screening, Metagenomics

## Abstract

*Historic Rebel* (*HR*) cheese is an Italian heritage cheese, produced from raw milk during the summer grazing period in the Alps. The aim of this work was (i) to characterize the cheese microbiota, by 16S rRNA gene amplicons sequencing, and the volatile and non-volatile lipophilic fraction, by Gas Chromatography and Dynamic Headspace Extraction-Gas Chromatography-Mass Spectrometry, and (ii) to evaluate their respective associations. *HR* cheese was dominated by Firmicutes phylum (99% of the entire abundance). The core microbiota was formed by *Streptococcus, Lactobacillus*, *Lactococcus*, *Leuconostoc* and *Pediococcus* genera together representing 87.2–99.6% of the total abundance. The polyunsaturated fatty acids composition showed a high PUFA n-3, PUFA n-6 and CLA content, two fold higher than typical plain cheeses, positively correlated with pasture altitude. A complex volatilome was detected, dominated in terms of abundance by ketones, fatty acids and alcohols. Total terpene levels increased at higher altitudes, being the main terpenes compounds α-pinene, camphene and β-pinene. The *HR* cheese showed a great diversity of bacterial taxa and lipophilic fractions among producers, despite belonging to a small alpine area, revealing a scarce cheese standardization and a chemical fingerprint of a typical mountain cheese produced during the grazing period. A deeper knowledge of the variability of *HR* cheese due to its composition in microbial community and volatile compounds will be appreciated, in particular, by elite consumers looking for niche products, adding economic value to farming in these alpine areas.

## Introduction

Cheese is a complex matrix that reflects the complexity of the environment in which is produced, especially in mountain areas. Microbial communities, as well as pasture grazing, contribute to its quality and physiochemical properties. The number of investigations on dairy product microbiota has increased in the last decade^1,2^, through the introduction of high-throughput DNA sequencing techniques (HTS), by 16S rRNA gene amplification, allowing deeper evaluations of the structure and the dynamics of microbial communities in foodstuff^3,4^. This approach has been used in several investigations of the microbial diversity of raw milk cheeses^5–7^, influenced by different factors, including animal feeding systems^8,9^, food processing environments^10^, and manufacturing techniques^11^.

Autochthonous bacteria, starter and non-starter lactic acid bacteria (NSLAB), originating from milk autochthonous bacteria and the environment, can modify the physical and chemical properties of cheese, throughout the whole production chain, during manufacture and ripening^2,12^.

The “Storico Ribelle” cheese (Historic Rebel: *HR*) is produced in the Orobic Alps (Central Alps, Lombardy region, Northern Italy), during summer pastures (Jun 1st–Sep 30th) at an altitude from 1500 to 2000 m a.s.l. and above, using raw cow milk (traditionally from the Italian Brown breed) with a supplement of 10–20% of fresh raw goat milk (Orobica breed). HR is a semi-cooked, semi-hard cheese, cylindrically shaped (40–50 cm diameter × 9–12 cm height) with concave sides and sharp edges, weighting 9–20 kg. The paste has a compact structure with sparse partridge-eye holes; the color varies from white to straw yellow according to the degree of ripening, from a minimum of 70 days up to 10 years or more. This cheese was initially called Bitto Storico to differentiate the cheese obtained strictly following the traditional recipe from Bitto DOP cheese, whose disciplinary allows to supplement cow diet with a maximum of 3 kg of dry matter per day, and to transform the milk with the use of indigenous starter cultures. A Consortium for the HR (https://www.formaggiobitto.com/en/) and a Slowfood presidium (https://www.fondazioneslowfood.com/en/slow-food-presidia/heritage-bitto/) were created to support *HR* production as gastronomic heritage considering the cultural, social, landscape, and eco-system aspects. The current production (as of 2020) is of about 2000 wheels per year.

The cheese-making process is linked to ancient local traditions: raw milk is processed in the *calècc*, traditional stone structures considered as itinerant dairies, one hour from milking using wooden equipment, considered essential to maintain the typical microbiota established in each alpine pasture (further information can be found in the *HR* website: https://www.formaggiobitto.com/en/storico/storico/production).

Since silage and diet supplementation, as well as the usage of starter cultures in cheese-making are prohibited by *HR* product specification, the high variability of organoleptic characteristics depends only on pasture composition and naturally occurring bacteria.

The absence of commercial starter cultures promotes the development of a natural cheese microbiota composed of bacteria, yeasts and moulds^5^. These NSLAB are a significant portion of bacteria in most cheese varieties during ripening and can also be used as microbial fingerprint^13^. In order to fulfill the nutritional needs of lactating cows, farmers use grazing techniques with herds in rotations from the lowest to the highest points of the alpine pasture following the phenological stage of herbs along the summer, helping fast pastures regeneration and guarantying high quality of grass.

During the summer season, in different areas, pastures are characterized by different fatty acid (FA) composition, as well as by a different terpenoid content. Mountain herbs contain a high level of polyunsaturated FA that are recognized as beneficial for human health^14^. Mountain pastures are also rich in terpenoids that possess an important olfactory impact that contribute to the “typicity” of mountain cheeses, and can also be used as markers of mountain production^15–17^. The resulting biodiversity of pastures, together with the microbiota of raw milk and of the environment, can lead to unique—complex—cheeses in terms of flavor and paste structure^18^. Variation of cheeses produced with the milk from different pastures and flowering periods is highly appreciated in particular by elite consumers.

However, the microbiota involved in *HR* cheese production has never been thoroughly assessed. This study aims to: (i) assess the characterization of the microbial variability of *HR* cheese by Next Generation Sequencing, and (ii) analyze the lipophilic fraction of the *HR* cheese, produced in different alpine pasture areas, to better characterize this heritage cheese in particular in those aspects linked to its production environment.

## Results and discussion

With the introduction of HTS techniques, several studies addressed the microbiota characterization of the major PDO cheeses, with the aim to improve their quality, safety and commercial values^1,2,19^. Scant information is available using HTS approach in traditional mountain cheese production linked to summer pastures biodiversity, including the Italian context^12,19^. For these reasons in this study, HTS and GC techniques were used for the first time as systemic approaches to characterize the biodiversity and richness of bacterial communities, the volatilome, terpenes and fatty acid profiles in *HR* cheese.

### LAB quantification through Real Time PCR

LAB quantification was performed in cheeses samples collected at 101 ± 4 days of ripening. For all the producers, the *Enterococcus* spp. formed the least abundant group among LAB bacteria, ranging from 10^6^ to 10^7^ CFU/g. *Leuconostoc* spp*.* and *Pediococcus* spp. levels ranged from 10^6^ to 10^8^ CFU/g. *Lactobacillus* spp. counts were from 10^7^ to 10^8^ CFU/g, while *Lactococcus* spp. levels ranged from 10^7^ to 10^9^ CFU/g, among producers. Finally, the *Streptococcus* spp. counts were equal to 10^8^ CFU/g, the prevalent group among LAB bacteria.

Our results were in line with the LAB levels reported both by Morandi et al.^20^ and by Colombo et al.^21^, in term of genera identification and quantitation, in Bitto PDO Italian cheese, a cheese produced with similar technology, in the same geographic area and in the same period of production of the *Historic Rebel*.

### Proximate analysis

The % of moisture ranged between 34.30 and 37.36 g/100 g, the % of protein ranged between 23.94 and 25.29 g/100 g and the % of fat between 31.11 and 34.33 g/100 g. No significant differences were detected among producers (Table 1). On the contrary, some significant differences were observed for moisture and fat across different grazing periods for the producers PP1, PP3 and PP4, with an increase of fat from July to September (Supplementary Files, Table S2).

**Table 1:** Proximate analysis mean values by Valley and Pasture area/Producer in % (g/100 g wb).

Valley	VG		VB		VA	
Pasture area-Producer	PP1	PP2	PP3	PP4	PP5	PP6
Moisture	34.30 ± 0.96	36.22 ± 0.55	37.55 ± 0.78	35.14 ± 0.69	37.36 ± 0.63	35.66 ± 0.59
Protein	25.24 ± 0.83	25.29 ± 0.48	24.74 ± 0.68	25.21 ± 0.60	23.94 ± 0.55	25.06 ± 0.51
Fat	34.33 ± 0.95	32.54 ± 0.55	31.11 ± 0.78	32.58 ± 0.69	32.43 ± 0.63	31.96 ± 0.59

*VG *Gerola valley, *VB *Brembana valley, *VA *Bitto di Albaredo valleys.

Data expressed as LSM ± SEM.

### Variability of the microbiota composition

HTS produced a total of 41,427,924 reads (avg. of 767,184 ± 240,765 per sample), which, after filtering, resulted in 24,388,097 reads (avg. of 451,631 ± 143,653 reads per sample). For computational reasons, a random subset of 100,000 reads was extracted from each sample. After de-noising and clustering, a high-confidence dataset of 2375 zOTUs (avg. of 1164 ± 192 zOTUs per sample) were obtained.

*Historic Rebel* cheese-associated microbiota was dominated by members of the Firmicutes phylum which accounted for about 99% of the entire abundance, with a minor presence of Proteobacteria. Carafa et al.^12^ and Dalmasso et al.^6^ noticed the same pattern in traditional mountain cheese produced with no starter strains in other alpine areas.

Generally, the overall bacterial composition of *HR* cheese showed a low diversity, with only 5 genera constituting the vast majority of the community: *Streptococcus* (mean relative abundance: 76.6%), *Lactobacillus* (mean rel. ab.: 10.5%), *Lactococcus* (mean rel. ab.: 5.9%), *Leuconostoc* (mean rel. ab.: 2.3%) and *Pediococcus* (mean rel. ab.: 2.1%) together represented an average of 97.2% (range among samples: 87.4–99.8%) of the total abundance. Only a minority of samples showed presence of *Escherichia* (7 samples, max. abundance 7.2%) and *Enterococcus* (11 samples, max. abundance 5.8%) (Supplementary Figure 1). As a general trend, triplicated samples per each GP- PP were correlated, as evident from unweighted Unifrac distances analysis, which showed a significant lower distances among replicated samples of the same PP-GP than that from samples of different PPs-GPs (P-value of Mann–Whitney *U* test: < 0.001) (Supplementary Figure 2).

**Figure 1:**
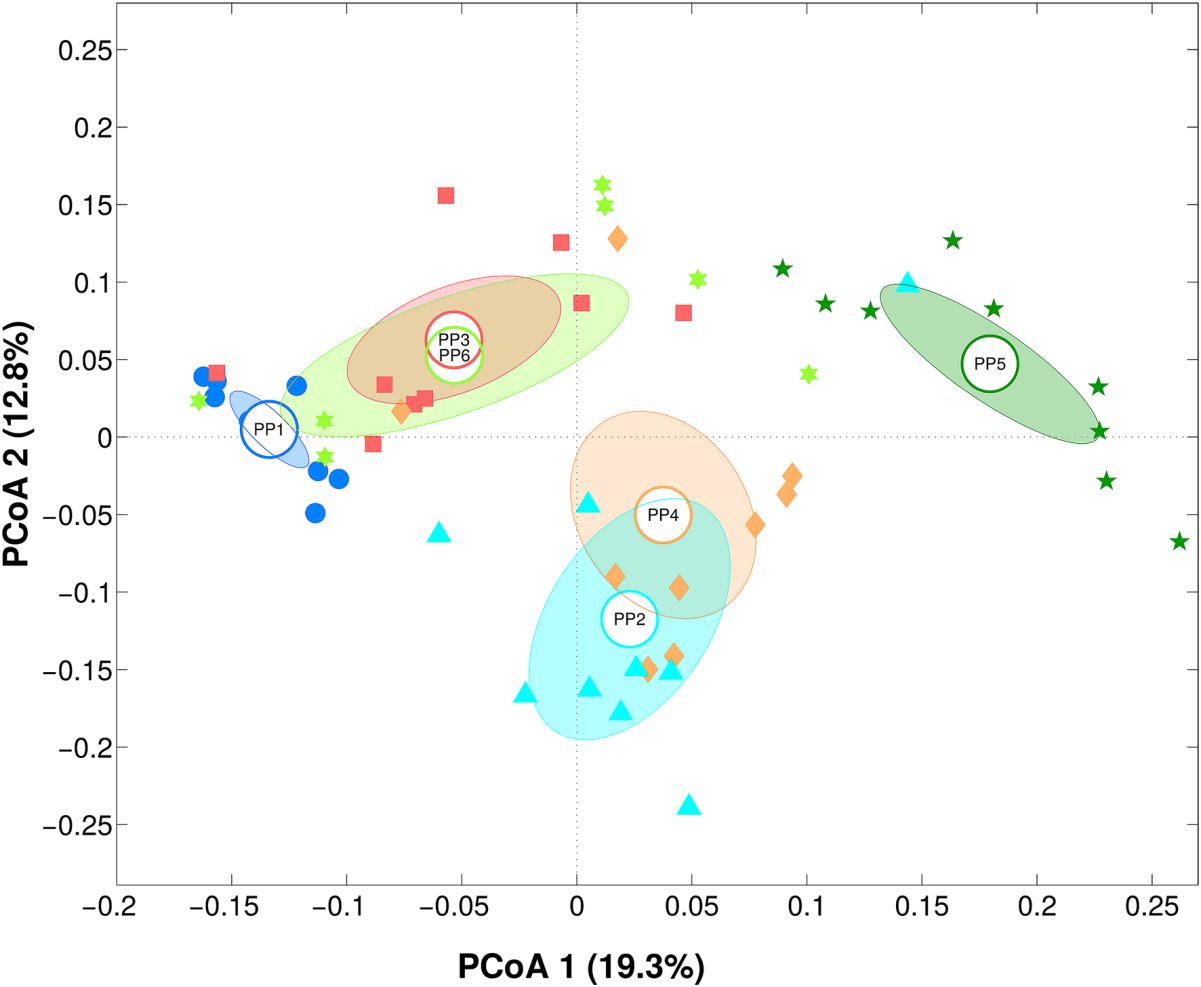
Principal Coordinate Analysis (PCoA) on unweighted Unifrac distances among samples grouped per Pasture area-Producer. Each dot represents a sample, the centroid represents its average value, and the ellipse the mean standard error (SEM) based data confidence. The first and second principal component is represented.

**Figure 2:**
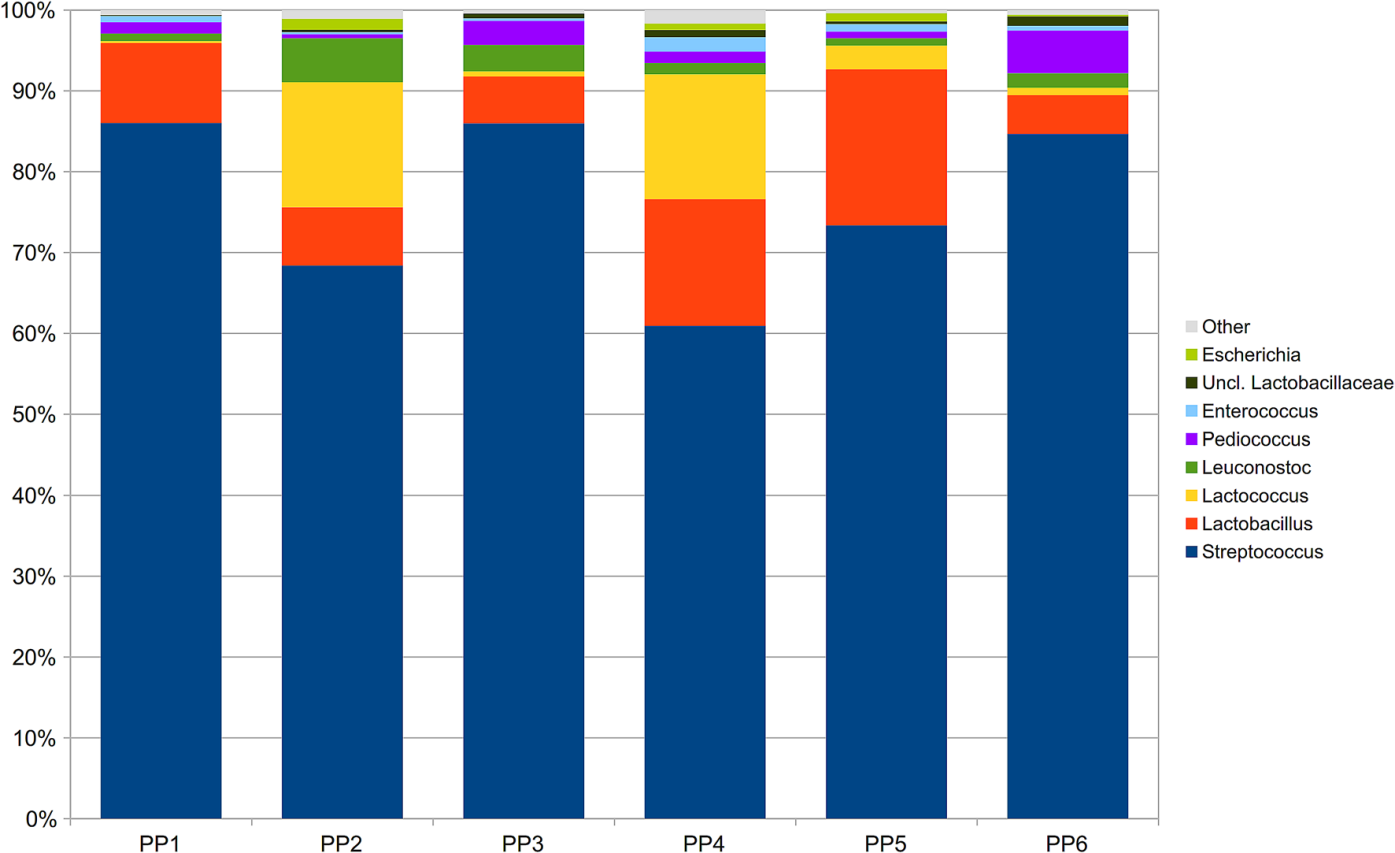
Stacked barplots depicting the average microbiota composition of *HR* cheese samples at genus level per each Pasture area-Producer. Data is presented as relative abundance on the overall microbial composition. Genera below 0.5% rel.ab. on average were grouped in “Other” category.

Analysis of the microbial community among GP-PPs, considering all time points and replicates together (n = 9), revealed how the cheese from each producer had a peculiar bacterial composition. They differed both on biodiversity (Kruskal–Wallis P-value < 0.01 on chao1, Shannon, observed species and Faith’s phylogenetic diversity metrics), with producer PP4 having the most biodiverse community and PP1 the least, and composition (P-value adonis test: 0.001, on both weighted and unweighted Unifrac distances). In particular, similar bacterial profiles were observed for PP2 and PP4, and for PP6 and PP3 (pairwise adonis test P-value > 0.05, weighted Unifrac distance), whereas PP5 and PP1 appeared to have a peculiar composition (pairwise adonis test P-value < 0.05 in all comparisons, weighted Unifrac distance). Taking into account also minor contributors of the microbiota, all GP-PPs were statistically different (P = 0.01, unweighted Unifrac distance) (Fig. 1). At genus level, *HR* cheese samples from PP2 were characterized by a somehow higher relative abundance of *Lactococcus* (15.5% on average) and *Leuconostoc* (5.4%); PP4 was characterized by a high rel. ab. of *Lactococcus* (15.4%), as well as the lowest presence of *Streptococcus* (60.9%); PP5 microbiota, instead, showed the highest rel. ab. of *Lactobacillus* (19.3%) and a subdominant presence of *Lactococcus* (2.9%). On the other hand, PP6, PP3 and PP1 samples were all characterized by higher percentages of *Streptococcus* (> 84.7%, whereas PP2, PP4 and PP5 were all < 74.0%), associated to *Pediococcus* in PP6 (5.3%), *Leuconostoc* in PP3 (3.3%) and *Lactobacillus* in PP1 (9.9%) (Fig. 2). On the other hand, comparing GPs, we observed a partial difference in alpha-diversity (Kruskal–Wallis P-value = 0.03 on Faith’s phylogenetic diversity metrics), due to a lower biodiversity in GPII; however, no difference was visible in composition (P-value adonis test > 0.05, on both weighted and unweighted Unifrac distances).

### Species-level analysis

**Figure 3:**
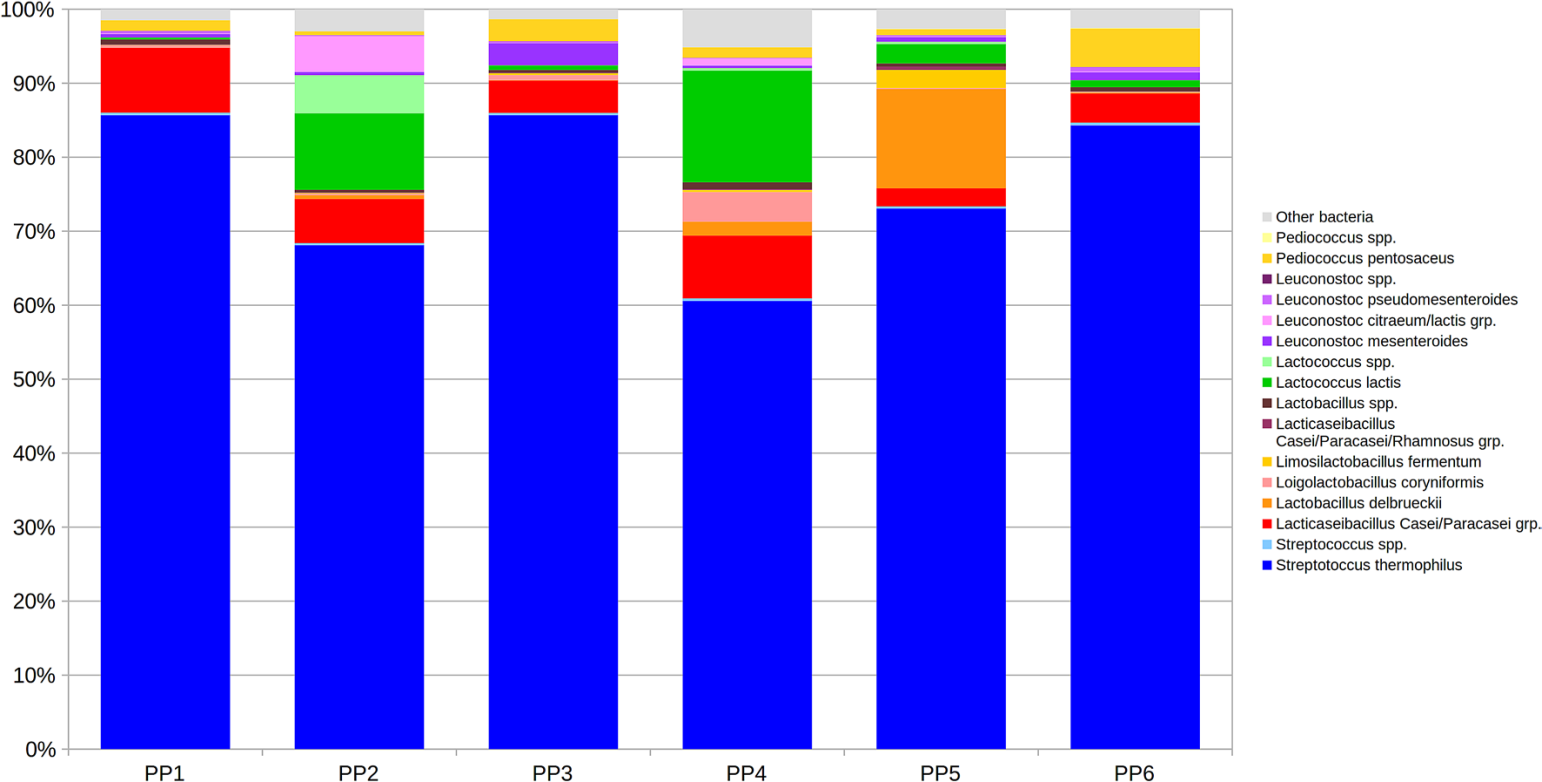
Stacked barplots depicting the average microbiota composition of *HR* cheese samples at species level per each Pasture area-Producer. Data is presented as relative abundance on the overall microbial composition. Only main species are represented. Low abundance species within each genus are grouped in the “spp.” record.

Since *Streptococcus*, *Lactobacillus*, *Lactococcus*, *Leuconostoc* and *Pediococcus* constituted more than 90% of the total bacterial population, a focus on these genera has been performed (Fig. 3 and Supplementary Figure 3). Species-level characterization identified a number of interesting features of the *HR* cheese microbiota coming from the six Pastures areas-Producers (PP).

Evaluating species composition within each genus allowed the identification of some peculiar traits of prevalence of certain species in determined PPs. Among *Streptococcus*, S. *thermophilus* represented most of the total, accounting for > 98% of total rel. ab. of this genus, on average. PP2 and PP4 samples only showed about 0.5% of other (unidentified) *Streptococcus*. These data confirm the findings of Morandi et al^20^, who reported the prevalence of *S. thermophilus* in Bitto DOP along with the presence in low numbers of *S. uberis, S. salivarius* and *S. macedonicus*. The presence of S. *thermophilus* in high numbers is a common trait in cooked cheeses where high temperatures are applied during cheese-making, as in HR cheese, and for this reason, S. *thermophilus* is generally used as starter. In addition, Cremonesi et al.^22^ found in milk from Rendena dairy cattle, a local Italian breed, at same farming conditions, a greater quantity of *S. thermophilus* in the local breed milk than in the mainstream breed Italian Holstein, indicating that the animal genetics may have an influence on the composition of the milk microbiota. The abundance of *S. thermophilus* in *HR* cheese could be also due to the use of typical wooden equipment during the cheesemaking process, from milk collection to ripening, with wooden poles, bands and shelves, acting as a possible natural reservoir of *S. thermophilus* strains of wooden vat origin, as demonstrated in other studies^23,24^, providing microbial diversity in dairy traditional products. The acidification of curd during cheese-making process, is probably mainly due to the presence of indigenous *S. thermophilus*, and is of paramount importance for the *HR* cheese production, where commercial starter cultures are not allowed. The other LAB species found in the Historic Rebel are almost the same as those found in previous studies regarding Bitto PDO^20,21^, confirming the close association between these two products. On the other hand, *Pediococcus pentosaceus* summed up to 98% rel. ab. of this genus, apart for PP6 and PP5 samples, for which 1.6% and 8.6%, respectively, was due to *P. acidilactici*. Among *Lactobacillus*, the majority of species were identified as *Lacticaseibacillus casei/paracasei* group. (accounting for about 66% of total rel. ab. on average), with some notable exceptions: PP5 showed 49.8% of *Lactobacillus delbrueckii* and 13.7% of *Limosilactobacillus fermentum*, whereas PP2, PP3 and PP4 were characterized by a somehow higher presence of *L. coryniformis* (rel. ab. of 6.9%, 13.5% and 21.9%, respectively). *Lactococcus* population was dominated by *L. lactis* with PP5, PP4 and PP2 showing the presence of other unidentifiable species (average rel. ab. 4.6%, 4.5% and 16.7%, respectively). Finally, among *Leuconostoc*, PP3 was composed for about 87% by *Ln. mesenteroides*, whereas PP1, PP5 and PP6 showed a consistent presence of *Ln. paramesenteroides* (average rel. ab. of 40.0%, 18.1% and 27.9%, respectively); PP2 and PP4, on the other hand, displayed a majority of reads belonging to *Ln. citreum/lactis* group.

With regard to species-level composition for the six Pastures areas-Producers (PP) (Fig. 3), on an absolute basis, *S. thermophilus* was the dominant species, as expected, with rel. ab. ranging from 60.6 to 85.7%. PP1 was composed quite entirely of *S. thermophilus* and members of *Lact. casei/paracasei* group. (together accounting for 94.5% of total rel. ab.); PP2 and PP4 were characterized by a higher presence of *L. lactis* (rel. ab of 10.3% and 15.1% compared to an average of 1.1% in other PPs; PP3 had the highest presence of *Ln. mesenteroides* (3.0%, compared to an average of 0.6%); PP5 showed a consistent presence (13.4%) of *L. delbrueckii*; finally, PP6 was composed for 5.2% of *P. pentosaceus* (compared to an average of 1.4% on other PP).

### Volatilome

Cheese volatilome represents the final step of catabolism of sugars, proteins and lipids by endogenous and microbial enzymes. Along with the terpenoid content, volatiles characterize the richness of flavor of mountain cheeses, highly appreciated by consumers.

The main volatile compounds detected in the headspace of the *HR* cheese were: 8 alcohols, 7 free fatty acids, 4 ketones, 3 esters, and 1 aldehyde (Supplementary Files Table S3a–d). Other components present at trace levels were disregarded. Among all classes of compounds, ketones represented the majority of the volatilome, followed by fatty acids and alcohols. Ketones and volatile free fatty acids derive from hydrolysis and subsequent β-oxidation of milk lipids promoted by the intracellular enzymes after bacterial cell lysis. Moreover, ketones have a strong impact on cheese flavor, because of their high odor strength, described as ethereal and sweet with a piquant note. In our study, the major detected ketones were: 3-hydroxy-2-butanone (acetoin), butan-2-one and pentan-2-one. The first two derive from the catabolism of citrate, important flavor compounds in several cheese varieties^25^. In particular, acetoin is responsible for the buttery notes in cheeses^26,27^. The predominance of ketones in volatilome was also detected by Panseri et al.^28^ and Povolo et al.^29^ in PDO Bitto cheese.

Fatty acids were the second most abundant compounds detected in *HR* volatilome. These carboxylic acids derive from the hydrolysis of triglycerides through the activity of endogenous and microbial enzymes, and are detectable in the headspace of the cheese only when the aliphatic chain is less than ten carbon number. They, also, contribute strongly to cheese flavor, like butanoic acid, characterized by a rancid cheese-like odor, which plays an important role in Camembert and Grana Padano cheeses flavor^30^. Fatty acids are precursors of other aroma compounds, such as methyl ketones, alcohols, lactones, aldehydes, and esters^31^. Branched-chain fatty acids like 3-methylbutanoic and 2-methylpropanoic acid, characterized by a “sweaty” and “ripened cheese” aroma, derive from isoleucine and leucine in the last steps of protein catabolism^27^ and were also found in PDO Bitto cheese^28^.

Ethyl butanoate, ethyl hexanoate and ethyl acetate were the most abundant esters isolated in the *HR* volatilome. They derive by the enzymatic esterification of free fatty acids, generally high in pasture-derived milk and cheese^32^. They play an important role in the formation of the fruity flavor in cheese and they are able to give this main fruity note to the flavor of some Italian cheeses^33^, being also the most potent odorants of Grana Padano, Ragusano and Cheddar^26,34^.

In our study, other important volatile compounds were the branched alcohols 3-Methylbutan-1-ol (medium odor strength, described as alcoholic, fuel), and Phenylethan-1-ol (medium odor strength, described as sweet almond, fresh), derived from the catabolism of amino acids leucine and phenylalanine, respectively^27^.

**Figure 4:**
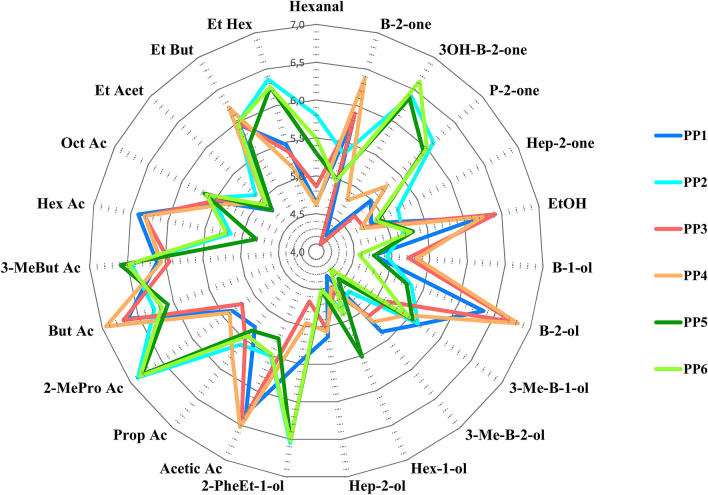
Radar plots showing the terpenoid profiles of *HR* cheese according to Pasture area-Producer. Abundance of terpenes is reported in arbitrary units as the logarithmic relative peak intensities of Quant_ion_.

Regarding the volatile metabolome as a whole, the comparison among the volatilomes of the six PPs considered, showed some similarities (Fig. 4). Some producers obtained cheeses characterized by a higher proteolysis, accompanied by a lower lipolysis like PP2, PP5 and PP6, (see the levels of 2-methyl propanoic acid and hexanoic acid respectively). The higher levels of ethanol from carbohydrates catabolism allowed the formation of ethyl esters from butanoic and hexanoic acid (from lipid catabolism), as for PP1, PP3 and PP4 that showed the higher levels of these volatiles. The odor strength of these esters is very high, and is described as fruity, sweet pineapple, very typical of mountain cheeses during grazing season.

**Figure 5:**
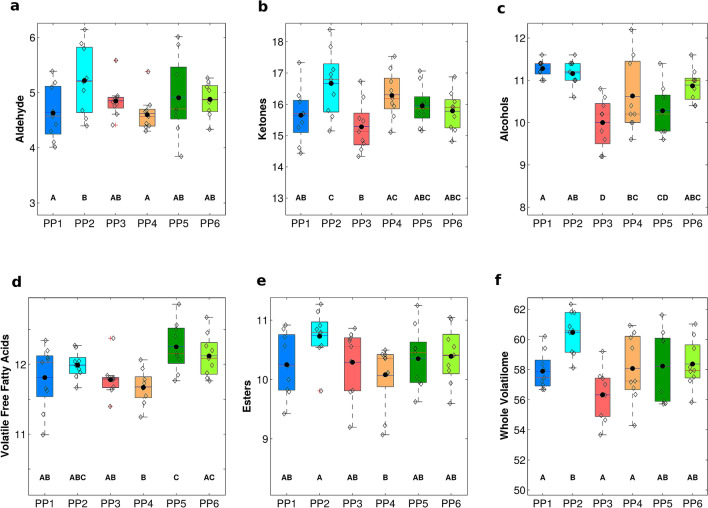
Box-plot of contents of the volatile compounds for each Pasture area-Producer: (**a**) aldehyde, (**b**) ketones, (**c**) alcohols, (**d**) volatile free fatty acids, (**e**) esters, (**f**) whole volatilome. Rhombus refer to *HR* cheese replicates. A mean value relative to cheese samples collected is indicated for comparison (dotted line). Data expressed as log_10_ of the area of the Quant_ion_ (LSM ± SEM). ^A–D^Values between the boxplot of each compounds with different superscript letters differ significantly at P < 0.01.

The differences in volatilome seem unrelated to the valley (Supplementary Files Table S3a–d). In fact, cheeses from the same valley, do not show common characteristics; therefore, the single producer has a paramount role in defining the cheese trait, both qualitative and quantitative. Moreover, within the single PP and the single period among producers (Fig. 5 and Supplementary Files Table S3a–d; respectively) different volatilomes were observed. Regarding the whole volatilome (Fig. 5), PP2 producer showed the maximum overall content. The complexity of *HR* volatilome, like in other mountain cheeses, derives, mainly, by the usage of raw milk, and by the complexity of the indigenous microbiota of that particular environment and of the whole cheese-making process, starting from feeding (grazing) till ripening in cellars.

### Volatilome and microbiome association

Since the microbial activity is of a paramount importance in producing volatiles, several studies were carried out investigating how the metabolism of different genera can contribute to cheese volatilome.

Overall, in our study, a positive association between some volatile compounds and the main bacterial genera has been found. In some cases, negative correlations were identified, not due to the absence of a bacterial species, which leads to an increase of some volatiles compounds, but by the fact that the decrease in the quantity of one bacterial group is associated with the presence of others, positively associated to some volatiles compounds.

**Figure 6:**
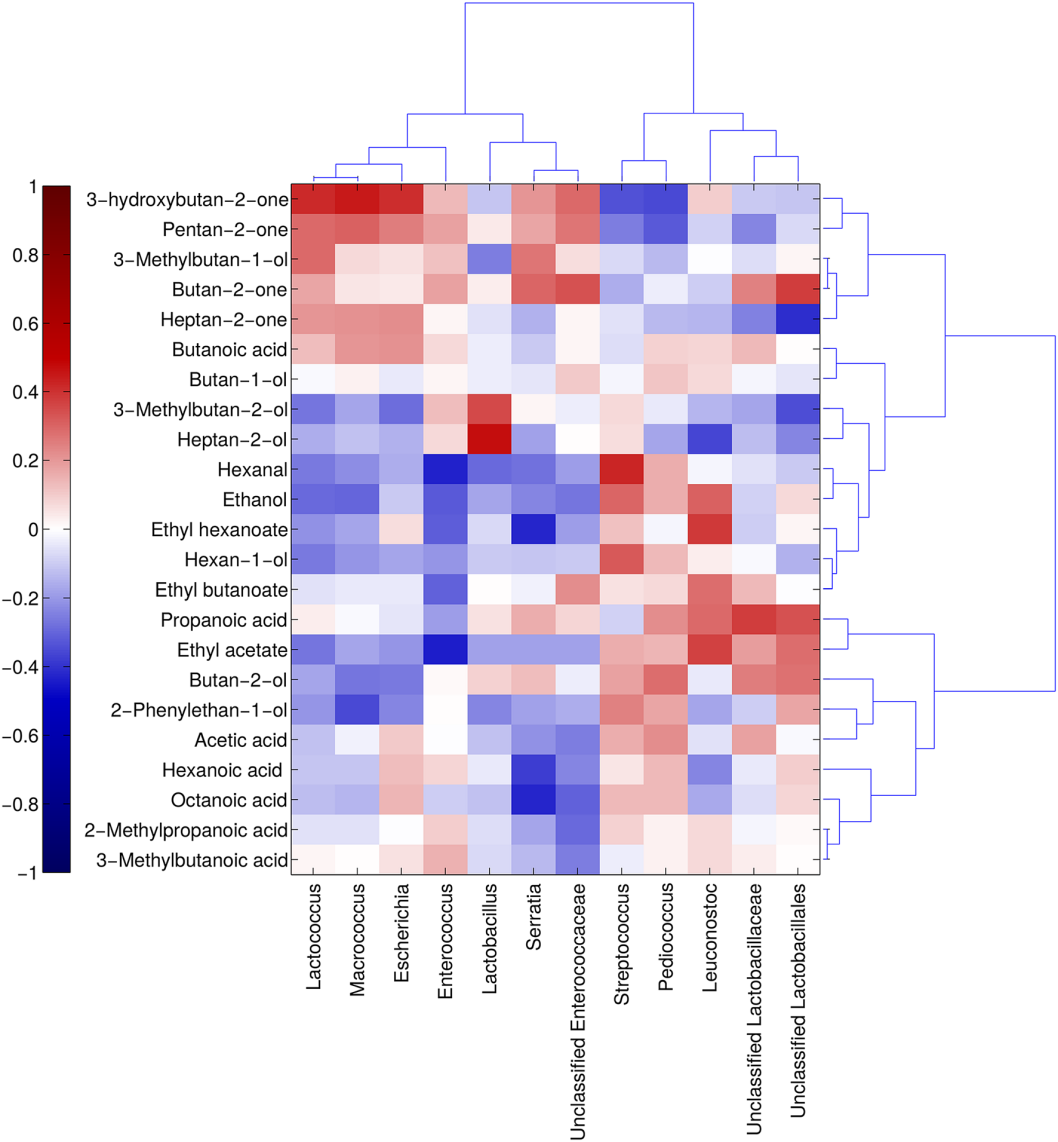
Heatmap plot reporting the Spearman's statistical correlation between the abundance of volatile compounds and the abundance of bacterial genera in the *HR* cheese samples. The color key is reported on the legend, with dark red (1) indicating a significant positive correlation (P < 0.05), and the dark blu (− 1) indicating a significant negative correlation (P > 0.05).

The volatiles that showed high and significant (P-value < 0.05) Spearman’s correlations with bacterial microbiome composition are reported in Fig. 6. Among the total volatiles, most ethyl-esters, along with ethanol, are correlated with *Leuconostoc,* as observed by Pogačić et al.^37^, confirming their potential combination as adjunct flavoring in cheese, associated with the development of fruity, sweet, and floral flavor notes, in particular in long ripened cheeses^30^. In our study the 3-Hydroxybutan-2-one (or acetoin, derived by citrate metabolism) is strongly and positively associated with *Lactococcus* genus*,* confirming the results of Pogačić et al.^35^ and of Gallegos et al.^36^ where 3-Hydroxybutan-2-one and 3-methylbutan-1-ol were positively correlated to *L. rhamnosus, L. paracasei, L. sakei* and *L. lactis* subsp *lactis, L. lactis* subsp. *cremoris, L. paracasei* subsp. *paracasei*. In our study 2-Heptanol, derived by the reduction of the corresponding ketone, showed a strong positive correlation with *Lactobacillus* as found by Dan et al.^37^, where this secondary alcohol was detected in samples fermented by different strains of *Lactobacillus*. Hexanal, a result of lipid oxidation, and its corresponding alcohol 1-hexanol, showed a strong positive correlation with *Streptococcus,* confirming the findings of Dan et al.^38^ where this compound was present in milk fermented by *S. thermophilus.* The only (fatty) acid that showed a significant positive correlation with *Leuconostoc* genus was propionic acid, derived by carbohydrate metabolism, as demonstrated by Porcellato et al.^39^ and Østlie et al.^40^, where *Leuconostoc* have been shown to dominate the cheese microbiota in the later stages of ripening with added propionic acid bacteria.

### Fatty acid composition

Among the three principal milk macro-components, protein, sugar and fat, the latter is the most susceptible to changes in composition. The most part of fatty acids, in fact, is not produced de novo in the mammary gland, but derives from the bloodstream, after the ruminal activity on feeding matter. When feeding changes, like along the summer grazing period, a change in fatty acid composition takes place. Several studies were carried out on this aspect, in particular with the aim to compare winter *versus* summer productions or mountain extensive production versus intensive indoor production^41–46^.

**Figure 7:**
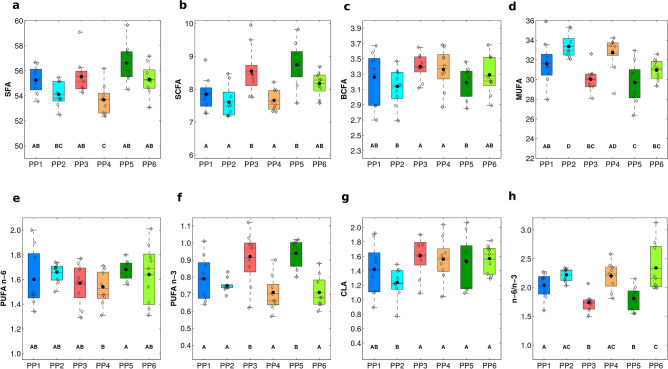
Box-plot of contents of the fatty acids composition for each Pasture area-Producer: (**a**) SFA, total saturated fatty acids; (**b**) SCFA, short chain fatty acids; (**c**) BCFA, branched chain fatty acids; (**d**) MUFA, total monounsaturated fatty acids; (**e**) PUFA-n6, total polyunsaturated fatty acids n-6; (**f**) PUFA-n3, total polyunsaturated fatty acids n-3; (**g**) CLA, conjugated linoleic acid; (**h**) ratio n6/n3. Rhombus refer to *HR* cheese replicates. A mean value relative to cheese samples collected is indicated for comparison (dotted line). Data expressed as least square mean ± standard error of mean. ^A–D^Values between the boxplot of each compounds with different superscript letters differ significantly at P < 0.01. Fatty acids were grouped in: SFA (C4 + C6 + C8 + C10 + C12 + C13 + C14 + C15 + C16 + C17 + C18 + C19 + C20), SCFA (C4 + C6 + C8 + C10), BCFA (iC14 + iC15 + aiC15 + iC17 + aiC17 + iC18), MUFA (C10:1 + C12:1 + C14:1 + C15:1 + C16:1 + C17:1 + C18:1 + C20:1), PUFA n-6 (C18:2c9,c12) PUFA n-3 (C18:2c9,c12,c15). CLA (C18:2c9,t11) and n6/n3 ratio was also considered.

The fatty acid composition (Fig. 7) for all the PPs reflects that of a typical mountain cheese produced during grazing period, the most important change being the higher amount of polyunsaturated fatty acids (Fig. 7e,f) and branched chain fatty acids (Fig. 7c) deriving from pastures rich in dicotyledons. The level of total CLA varied from 1.06 to 1.85% (Supplementary Files Table S4), in agreement with data reported for other PDO cheeses produced during grazing period^15,17,46^, when no maize silage is given to cattle. Mean value for CLA (% FA) obtained in our study is more than twice (overall Least Mean Square ± Standard Error Means, 1.48 ± 0.05, Fig. 7g) respect to plain cheeses, where cattle are fed concentrates^45^. For the same reason, similar findings were noticed for BCFA (overall LMS ± SEM, 3.27 ± 0.05, (Fig. 7c) and n-3 PUFA (overall LMS ± SEM, 0.80 ± 0.05, Fig. 7e,f), confirming data of other authors^47,48^.

Among producers, PP3 and PP5 showed a higher abundance of SFA and PUFA, and a lower abundance of MUFA (Fig. 7a,e,f,d; respectively). Since, during the production season, herds move to different grazing areas at diverse altitudes, characterized by different composition and phenological stage of herbs, a significant difference in the fatty acid composition among the PPs (Fig. 7) and within the same PP during the three grazing periods (Supplementary Files Table S4) was detected. Producer PP3 that grazed the herd at higher altitudes produces cheeses with a higher content of CLA and a lower n6/n3 ratio (Fig. 7g,h). The PP1, PP5 and PP6 showed huge variability among different GPs (Supplementary Files Table S4), indicating that their pastures differed during the summer season, especially from GPI and GPII, instead PP2 and PP3, showed less variability. PP5 and PP6 belonged to the same valley, the Bitto di Albaredo valleys (VA).

### Terpenoids composition

A clear distinctive character between mountain and plain vegetation is the content of terpenoids, an important class of compounds derived by isoprene units emitted by mountain herbs, mainly dicotyledons, in defense to predators. Dicotyledons are the main constituents of summer pasture and, among these, Apiaceae, Lamiaceae, and Asteraceae families contain high levels of terpenoids, while Poaceae along with Fabaceae, mainly constituents of the indoor feeding from plain, do not^49^. Because of this, terpenoids have been proposed as potential pasture-diet marker^16^. Moreover, they can contribute floral and vegetal odor to the flavor of cheese made with milk from grazing animals^49^. Ten terpenoids resulted statistically different (P < 0.01) among the PPs (Table 2). A similar composition was noticed in other PDO mountain cheeses as “Fontina Valle d’Aosta” and “Bitto”^16,17,28^. The richness (both qualitatively and quantitatively) of terpenoid moiety found in all the *HR* cheese samples, is typical of a mountain production during summer grazing period, where these volatile lipophilic compounds are transferred to the milk and finally to the cheese^49^. α-Pinene, camphene and β-pinene were the most abundant compounds in *HR* cheeses. Their presence is confirmed in many plants from alpine pasture^17,49^.

**Table 2:** Main terpenoids identified in *Historic Rebel* cheese according to Valley and Pasture area/Producer**.**

Valley	VG^1^		VB		VA	
Pasture area-Producer	PP1	PP2	PP3	PP4	PP5	PP6
*α-*Pinene	6.45 ± 0.12^A^	6.52 ± 0.12^A^	6.73 ± 0.12^A^	6.32 ± 0.12^A^	7.24 ± 0.12^B^	6.56 ± 0.12^A^
camphene	5.42 ± 0.09^A^	5.17 ± 0.09^AB^	5.17 ± 0.09^AB^	4.98 ± 0.09^B^	5.80 ± 0.09^C^	5.16 ± 0.09^AB^
*β*-Pinene	5.74 ± 0.10^AB^	5.81 ± 0.10^AB^	5.73 ± 0.10^AB^	5.45 ± 0.10^B^	6.02 ± 0.10^A^	5.57 ± 0.10^B^
δ-3-Carene	5.18 ± 0.13^AB^	4.94 ± 0.13^A^	5.72 ± 0.13^C^	5.46 ± 0.13^BC^	5.42 ± 0.13^ABC^	5.00 ± 0.13^AB^
Sabinene	4.57 ± 0.11^A^	4.60 ± 0.11^A^	4.91 ± 0.11^AB^	4.65 ± 0.11^A^	5.20 ± 0.11^B^	4.63 ± 0.11^A^
Limonene	5.15 ± 0.09^A^	5.16 ± 0.09^A^	5.39 ± 0.09^A^	5.12 ± 0.09^A^	5.75 ± 0.09^B^	5.19 ± 0.09^A^
*γ*-Terpinene	4.83 ± 0.10^A^	5.02 ± 0.10^AB^	4.91 ± 0.10^AB^	4.78 ± 0.10^A^	5.23 ± 0.10^B^	4.86 ± 0.10^A^
α-Terpinolene	4.35 ± 0.11^A^	4.28 ± 0.11^A^	4.56 ± 0.11^AB^	4.40 ± 0.11^AB^	4.78 ± 0.11^B^	4.37 ± 0.11^A^
*allo*-Ocimene	4.84 ± 0.11^A^	4.89 ± 0.11^A^	5.11 ± 0.11^A^	4.70 ± 0.12^A^	5.61 ± 0.11^B^	4.93 ± 0.11^A^
*β*-Caryophyllene	4.90 ± 0.10^A^	5.09 ± 0.10^AB^	5.40 ± 0.10^BC^	5.19 ± 0.10^AB^	5.55 ± 0.10^B^	4.98 ± 0.10^A^

*VG *Gerola valley, *VB *Brembana valley, *VA *Bitto di Albaredo valleys.

Data expressed as the log10 of the area of the quant ion (LSM ± SEM).

^A,B,C^Values within a row with different superscript letters differ significantly at P < 0.01.

In our study, a difference in terpenoid levels was detected among PPs (Table 2), suggesting that these compound can discriminate productions between different pastures^15^. Panseri et al.^28^ in a study comparing cheeses from different producers of PDO Bitto, the neighborly produced cheese, didn’t found such difference. The producers PP3, PP5 and PP6, settled at the highest grazing areas (2000, 1950, 1930 m a.s.l., respectively), produce cheeses characterized by the higher levels of total terpene contents. Among PPs, PP5 showed the highest abundance of terpenoids. The terpenoids that best differentiated the PPs were *allo*-ocimene, α-terpinolene, α-pinene, and δ-3-carene. The latter, especially, was directly related to the altitude of grazing within the same producer PP5 (Supplementary Files Table S5).

## Conclusions

This study, by using a 16S rRNA-based amplicon sequencing approach for microbiota profiling, associated to volatilome, terpenes and fatty acid profiles, allowed a deep characterization of the Historic Rebel, a raw milk cheese produced in a limited alpine area.

This study allowed to identify distinctive characters of HR regarding cheese core microbiota that is closely correlated with the profile of VOCs, the profile of fatty acids, closely related with pasture feeding and also the identification of terpenoid compounds that depend on pasture vegetation and have a positive impact on the flavor of the cheese. Very in-depth information was also provided in relation to the variability of these characteristics according to producers, pasture areas, altitude and grazing period.

For these reasons, certain producers during the different production periods can offer to the consumer a particular cheese, characterized by its own microbiota, and enriched by the herbs of its particular pasture, with higher levels of PUFA and CLA and lower n6/n3 ratio.

It was demonstrated that the single pasture area—producer have a paramount role in defining the Historic Rebel cheese’s microbiota, volatilome, fatty acid profile and terpenoids composition, making this cheese a stronghold of the Orobic Alps biodiversity.

The associations observed among volatilome, and bacterial microbiome provide a basis for further studies in other areas of the Alpine Arch. Some producers are already aware of their role in maintaining elements of the rural culture of the area, including the typical itinerant cheese dairies, named *calècc* and the wooden tools for cheese making. Beside further understanding of the chemical fingerprint of the cheese, future research should widen the picture by addressing the importance of preserving the cultural and environmental values associated to the Historic Rebel cheese, and to identify strategies to convert these values into the economic sustainability of the *HR* Consortium.

## Material and methods

### Experimental design and sampling

*Historic Rebel* cheese, from six out of the twelve producers of the *HR* Consortium, located in the Orobie Alps (Central Italian Alps) in three different valleys, Gerola valley (VG), Brembana valley (VB), and Bitto di Albaredo valleys (VA), were analyzed. The six producers, two from each valley, corresponded to six different alpine pastures at straight-line distances among them of ≥ 12 km, (mean pasture area: 2.36 km^2^, 1.03–5.00), identified as PP1 to PP6 (Pasture area-Producer). Three grazing periods (GP), at the beginning (GPI, 23 days), in the middle (GPII, 20 days), and at the end (GPIII, 20 days) of the summer production season, from Jun 1st to Sep 30th 2015, were analyzed. For additional information on the experimental design, please, refer to Supplementary Files (Table S1).

A total of 54 samples of *HR* cheese ripened for 101 ± 4 days (range 93–106) were collected, equally distributed among the six Pasture area—Producers (PP1–PP6) and the three grazing periods (GPI–GPIII). Three cheese replicates from independent edges were collected in each week of sampling. A slice of cheese (approximately 250 g) representative of the whole wheel (from the rid to the center) was obtained by means of a sterile knife. Cheese samples were immediately vacuum-packed in polyethylene bags, stored at − 18 °C, and thawed at 20 °C before analysis. Each cheese slice was grated to obtain a homogeneous sample and the total amount was divided into different portions for the different analyses.

### LAB quantification through Real Time PCR

The Real Time PCR approach was used to quantify the LAB in the samples. *Lc. lactis* subsp. *lactis* MF 118, *L. delbrueckii* subsp. *bulgaricus* MF117, *St. thermophilus* MF69A, *Ln. mesenteroides* MF20, *Pd. pentosaceus* BT154, *E. faecium* RAF81 strains were provided by the ISPA-CNR (Institute of Sciences of Food Production-Italian National Research Council, Milan, Italy) bacterial collection and they were used for the standard curves.

The protocol described in Cremonesi et al.^50^ was used, without the pre-treatment step, to extract the DNA starting from 1 mL of overnight reference cultures (approximately 1–3 × 10^9^ CFU/mL). Then, the DNA was ten-fold diluted and used for the set-up of the standard curve (3 points in triplicates); in each assay a negative control was also included. Each sample was tested in triplicates.

Based on published 16S DNA sequences available in GenBank database (https://www.ncbi.nlm.nih.gov/genbank/), the primer pairs specific for *Lactobacillus* spp. (LACTOB-F: 5′-GAAGAGGACAGTGGAACTCCATGT-3′, LACTOB-R: 5′-CGCCACTGGTGTTCTTCCAT-3′), *Lactococcus* spp. (LACTOC-F: 5′-CGAGCGCAGGTGGTTTATTAA-3′, LACTOC-R: 5′-TTCCAATGCATACAATGGTTGAG-3′), *Streptococcus* spp., (STREP-F: 5′-CGGGTGAGTAACGCGTAGGT-3′, STREP-R: 5′-GCGGTATTAGCTATCGTTTCCAA-3′), *Leuconostoc* spp. (LEUCON-F: 5′-TGTCGCATGACACAAAGTTAAAAG-3′, LEUCON-R: 5′-CACCGCGGATCCATCTCTA-3′), *Pediococcus* spp. (PEDIOC-F: 5′-GCTTGCACTGAATGAGATTTTAACA-3′, PEDIOC-R: 5′-CTGCTTCTGGGCAGGTTACC-3′) and E*nterococcus* spp. (ENTA2-F: 5′-CTGTTGTTAGAGAAGAACAAGGA-3′, ENTA2-R: 5′-CGCTTTACGCCCAATAAATCC-3′), and were designed using Primer Express software (version 3.0) running standard settings. The primers were synthesised by Eurofins Genomics GmbH (Ebersberg, Germany) and were resuspended to a final concentration of 100 pmol/μl in sterile double-distilled water.

The Real Time reaction mixture, performed in a final volume of 10 μl, included 4 μl 1:10 dilution of the DNA as template, 1 ×. Power SYBR Green Master Mix (Applied Biosystems, Foster City, CA, USA) and 0.5 μM of each primer. The analysis was carried out on QuantStudio6 Flex Real-Time PCR System (ThermoFisher Scientific, Carlsbad, CA, USA) with a standard programme (50 °C for 2 min/95 °C* for 10′/40 cycles 95 °C* for 15″ and 60 °C* for 1′).

### Proximate analysis

Cheese samples were analyzed for moisture, protein, and fat content, at ARAL (Associazione Regionale Allevatori della Lombardia, Crema, Italy) laboratories. Moisture was determined by gravimetric method (ISO 5534:2004), protein was determined as total nitrogen × 6.38 (ISO 8968-1:2014), fat content by Rose-Gottlieb Method (ISO 1735:2005).

### DNA extraction and purification

For the metagenomics analysis, 45 mL of 2% (w/v) K_2_HPO_4_ buffer solution (Sigma-Aldrich, St. Louis, MO, USA) were added to 5 g of each sample; the sample was, then, mixed for 1 min and 30 s in a Stomacher machine (PBI, Milan, Italy). The DNA was extracted starting from 800 µL of the homogenized sample following the protocol described in Cremonesi et al.^50^ with some modifications. Briefly, 400 μL of lysis buffer (3 mol/L guanidine thiocyanate, 20 mmol/L EDTA, 10 mmol/L Tris–HCl, pH 6.8, 40 mg/mL Triton X-100, 10 mg/mL dithiothreitol) and 300 μL of binding solution (40 mg/mL silica from Sigma Aldrich, directly suspended in the lysis buffer) were added to the sample and vortexed for 30 s to obtain an emulsified solution. Then, the sample was incubated for 5 min at room temperature. After centrifugation for 30 s at 550×*g*, the supernatant was discarded and the silica-DNA pellet obtained was subsequently washed twice with 500 μL of lysis buffer, twice with 500 μL of washing solution (25% absolute ethanol, 25% isopropanol, 100 mmol/L NaCl, 10 mmol/L Tris–HCl, pH 8) and once with 500 μL of absolute ethanol. After every washing and vortexing, the silica-DNA pellet was centrifuged for 30 s at 550×*g* and the supernatant was discarded. The pellet was then vacuum-dried for 10 min. After addition of 100 μL of elution buffer (10 mmol/L Tris–HCl, pH 8, 1 mmol/L EDTA), the silica-DNA pellet was gently vortexed and incubated for 10 min at 65 °C. After a 5 min centrifugation at 550×*g*, the supernatant containing the DNA was recovered and stored at − 20 °C. DNA concentration and purity were measured using a NanoDrop ND-1000 spectrophotometer (NanoDrop Technologies, Inc., Wilmington, DE, USA).

### Preparation of library of the 16S rRNA gene and sequencing

Genomic DNA extracted from cheese samples was amplified using primers described by Caporaso et al.^51^, targeting the V3–V4 hypervariable region of the 16S rRNA gene. All PCR amplifications were performed by using 1 × HotStarTaq Master Mix (Qiagen, Hilden, Germany) following manufacture instructions, with 0.2 µL of each primer (100 µM) and 2 µL of genomic DNA (5 ng/µL). Amplicon cycling conditions were 15 min at 94 °C, followed by 25 cycles of 1 min at 94 °C, 1 min at 58 °C and 1 min at 72 °C, and finally 7 min at 72 °C. Then amplicons were cleaned-up with Agencourt AMPure XP (Beckman Coulter, Brea, CA, USA) following 16S Metagenomic Sequencing Library Preparation protocol (Illumina, San Diego, CA, USA) and checked for amplicons size with 2100 Bioanalyzer Instruments (Agilent Technologies, Santa Clara, CA, USA). Libraries were prepared by a second PCR amplification step using Nextera XT Index 1 Primers FC-131-1002, following 16S Metagenomic Sequencing Library Preparation protocol. The libraries obtained were quantified by Real Time PCR with KAPA library Quantification Kits (KapaBiosystems, Inc. MA, USA) pooled in equimolar proportion and sequenced on a paired 2 × 250 bp run on a Miseq platform (Illumina).

### Volatilome analysis

The volatilome produced by bacteria during cheese ripening was determined by Solid Phase Micro Extraction-Gas Chromatography-Mass Spectrometry (SPME-GC-MS) on 2 g of finely grinded cheese by means of a Combi-Pal automated sampler (CTC Analytics AG, Zwingen, Switzerland) coupled to an Agilent 6890 gas chromatograph with an Agilent 5975 mass spectrometric detector (Agilent Technologies) and a polar column (60 m × 0.25 mm × 10.25 µm, Zebron ZB-WAX plus, Phenomenex, Torrance, CA, USA). Extraction and separation conditions are described elsewhere^52^. Results are expressed log_10_ of the peak area (arbitrary units) of the corresponding selected ion.

### Terpenoid analysis

The terpene fraction was quantified by Dynamic Headspace-Gas Chromatography-Mass Spectrometry (DHS-GC-MS) on 2 mL of melted cheese fat obtained without the use of solvents, by centrifugation at 49,000×*g*, per 1 h at 40 °C, as described in De Noni and Battelli^16^. Briefly, terpenoid compounds were extracted from the head space of the melted fat by means of a DHS Master (Dani Instrument, Cologno Monzese, Italy) with 500 mL of high grade purity nitrogen during 18 min, trapped on a Tenax-TA trap (270 mg) at 40 °C, and directly desorbed at 260 °C for 3 min in the injection port of the same gas chromatograph, equipped with the same column, used for volatilome determination^17^. Results are expressed log_10_ of the peak area (arbitrary units) of the corresponding selected ion.

### Cheese fatty acid analysis

Fatty acid (FA) profile was determined on the same melted cheese fat obtained without the use of solvent for terpene determination. The FA methyl esters in hexane were injected into a gas chromatograph (GC) Agilent 7890 GC system (Agilent Technologies) equipped with on-column injector, and an FID detector. The separation was performed on a 100% dimethylpolysiloxane column (CP-Sil88 for FAME, 100 m × 0.25 mm × 25 μm). Derivatization and chromatographic condition were previously described^45^.

### Statistics and bioinformatics data analysis

Statistical analyses for chemical and chromatographic data were performed by using the SAS package v 9.4 (SAS Institute Inc., Cary, NC, USA). The two-way analysis of variance procedure (PROC GLM) was used to analyze the effects of alpine pasture area, grazing periods and the percentage of goat milk added on the variables of the proximate analysis, volatile organic compound evaluation, terpene and cheese fatty-acid profile. The model included as fixed effects the interaction between alpine area and grazing period and the percentage of goat milk nested in the Pasture area/Producer (PP1–PP6). Altitude was considered as covariate. Results are given as adjusted least squares means ± standard error means (LSM ± SEM).

For microbiota analyses, raw sequencing reads were processed performing a local assembly between two overlapping pairs, generating a single fragment covering the whole V3–V4 amplicon, using PandaSeq software^53^ (v2.5, “PAired-eND Assembler for DNA sequences”), and discarding those of length outside 250–900 bases, as well as non-overlapping sequences. Sequences having more than 25% nucleotides with a Phred score of 3 or less were filtered out using the “split_libraries_fastq.py” utility of the QIIME suite^54^ (release 1.8.0). Filtered reads were de-duplicated and de-noised creating zero-radius Operational Taxonomic Units (zOTUs) by unoise3 algorithm^55^ in usearch (v. 11.0.667), discarding those with less than 5 supporting reads, and taxonomically classified against the 13.8 release of the Greengenes bacterial 16S rRNA database (ftp://greengenes.microbio.me/greengenes_release/gg_13_8_otus) by RDP classifier^56^ at 50% confidence. Taxonomic characterization at species-level was performed by BLAST-aligning all reads belonging to five selected genera (i.e.: *Streptococcus*, *Lactobacillus*, *Lactococcus*, *Leuconostoc* and *Pediococcus*) to a custom reference database made up collecting all available reference sequences in NIH-NCBI database (ftp://ftp.ncbi.nlm.nih.gov/genomes/refseq/bacteria/) for a total of 7009 strains belonging to 232 species. Potential matches were filtered to retrieve an unequivocal classification for each read. Additional details on species-level classification can be found in Supplementary Methods. As of April 2020, genus *Lactobacillus* underwent major re-classification into 25 different genera, including 23 novel^57^. Taxonomy classification in this paper was performed according to the previous classification due to how reference databases were built. In species-level characterization, however, species have been named, where needed according to the new nomenclature. Sample biodiversity (i.e.: α-diversity evaluation) was estimated according to different microbial diversity metrics (i.e.: chao1, Shannon index, observed species and Faith’s phylogenetic distance). Inter-sample diversity (i.e.: β-diversity) analysis was conducted using both weighted and unweighted Unifrac metrics^58^ and Principal Coordinates Analysis (PCoA). Data separation was assessed with a permutation test with pseudo F-ratios using “adonis” function from R package^59^ “vegan” (version 2.0-10). For relative abundance analysis, a Mann–Whitney *U* test was used. Volatile compounds (VOC) quantities were correlated to bacterial composition by calculating Spearman’s correlation coefficient between normalized VOC quantities (both on sample and on single VOC basis) and bacterial genera present ≥ 1% rel. ab. in at least 1 sample; only correlations with a statistically non-zero coefficient were considered. For each statistical analysis, unless otherwise stated, P-values < 0.05 were considered as significant. All statistical evaluations were performed in Matlab (Software version 7.7.0, Natick, MA, USA).

## Supplementary Information

Supplementary Figures.

Supplementary Tables.

Supplementary Information.

## Data Availability

Raw reads of 16S rRNA amplicons of HR microbiome samples and related BioProject generated and analysed during this study are available in NCBI Short Read Archive repository (SRA, http://www.ncbi.nlm.nih.gov/sra) under accession number PRJNA644819.
