# Supplementary material for: High biodiversity in a limited mountain area revealed in the traditional production of Historic Rebel cheese by an integrated microbiota–lipidomic approach
Source: Sci Rep. 2021 May 14;11:10374. doi: 10.1038/s41598-021-89959-x (PMC8121794; doi:10.1038/s41598-021-89959-x)
Supplement: Supplementary file 1 — Supplementary Figures. [file 41598_2021_89959_MOESM1_ESM.pdf]

High biodiversity in a limited mountain area revealed in the traditional production of Historic Rebel cheese by an integrated microbiota-lipidomic approach

Federica Turri[1, +,@], Paola Cremonesi[1, +], Giovanna Battelli[2], Marco Severgnini[3], Milena Brasca[2], Gustavo Gandini[1,4], Flavia Pizzi[1]

[1] Institute of Agricultural Biology and Biotechnology, National Research Council, via Einstein, 26900 Lodi, Italy.

[2] Institute of Sciences of Food Production, National Research Council, via G. Celoria 2, 20133 Milan, Italy.

[3] Institute of Biomedical Technologies, National Research Council, via Fratelli Cervi 93, 20090 Segrate, Italy.

[4] Department of Veterinary Medicine, University of Milan, via dell'Università 6, 26900 Lodi, Italy.

[+] **These authors contributed equally to this work.**

[*] **Corresponding author, postal address: Agricultural Biology and Biotechnology, National Research Council, Street Einstein, 26900, Lodi, Italy; e-mail address: federica.turri@ibba.cnr.it**

Supplementary Figures

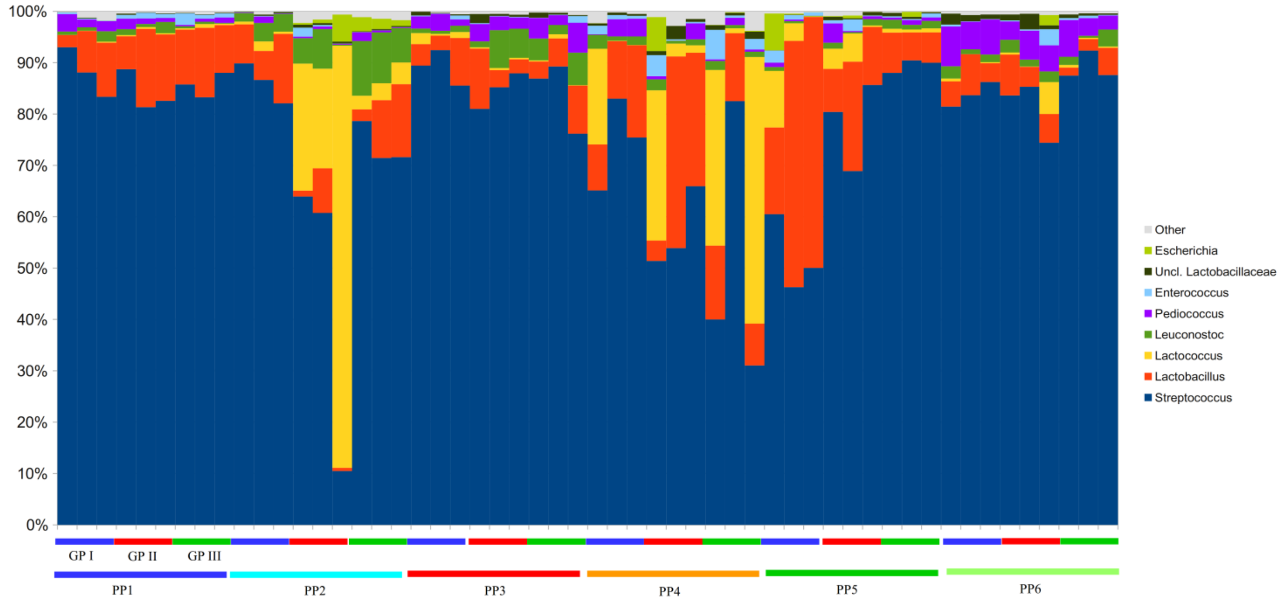

Supplementary Figure 1. Stacked barplots depicting the microbiota composition of *HR* cheese samples at genus level for each replicate. Data is presented as relative abundance on the overall microbial composition. Data are grouped according to PP and GP. Colored bars under the plots define the replicated samples. Genera below 0.5% rel.ab. on average were grouped in “Other” category.

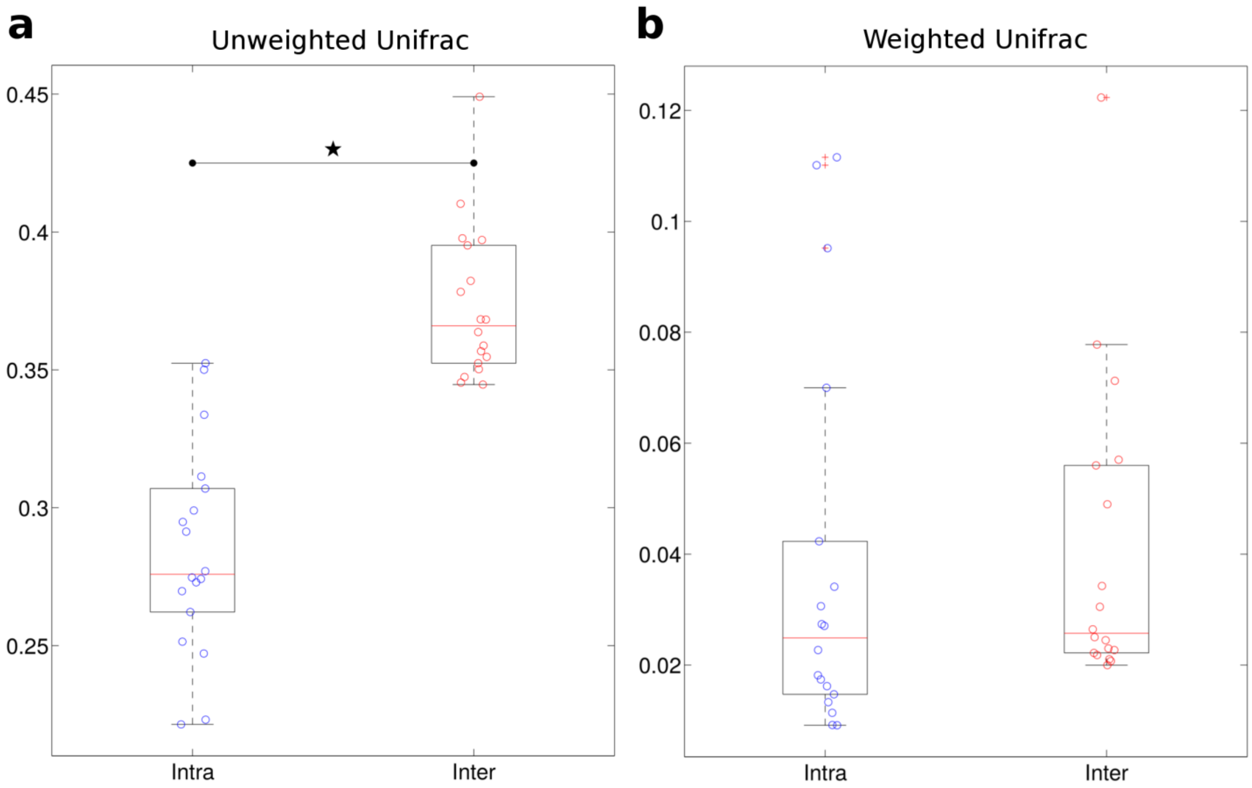

Supplementary Figure 2. Boxplots of intra- and inter-sample distances for (**a**) unweighted Unifrac and (**b**) weighted Unifrac distances calculated on replicated samples from the same Pasture area-Producer and Grazing Period (n=3). Significant differences (p-values <0.05) are highlighted with a ★.

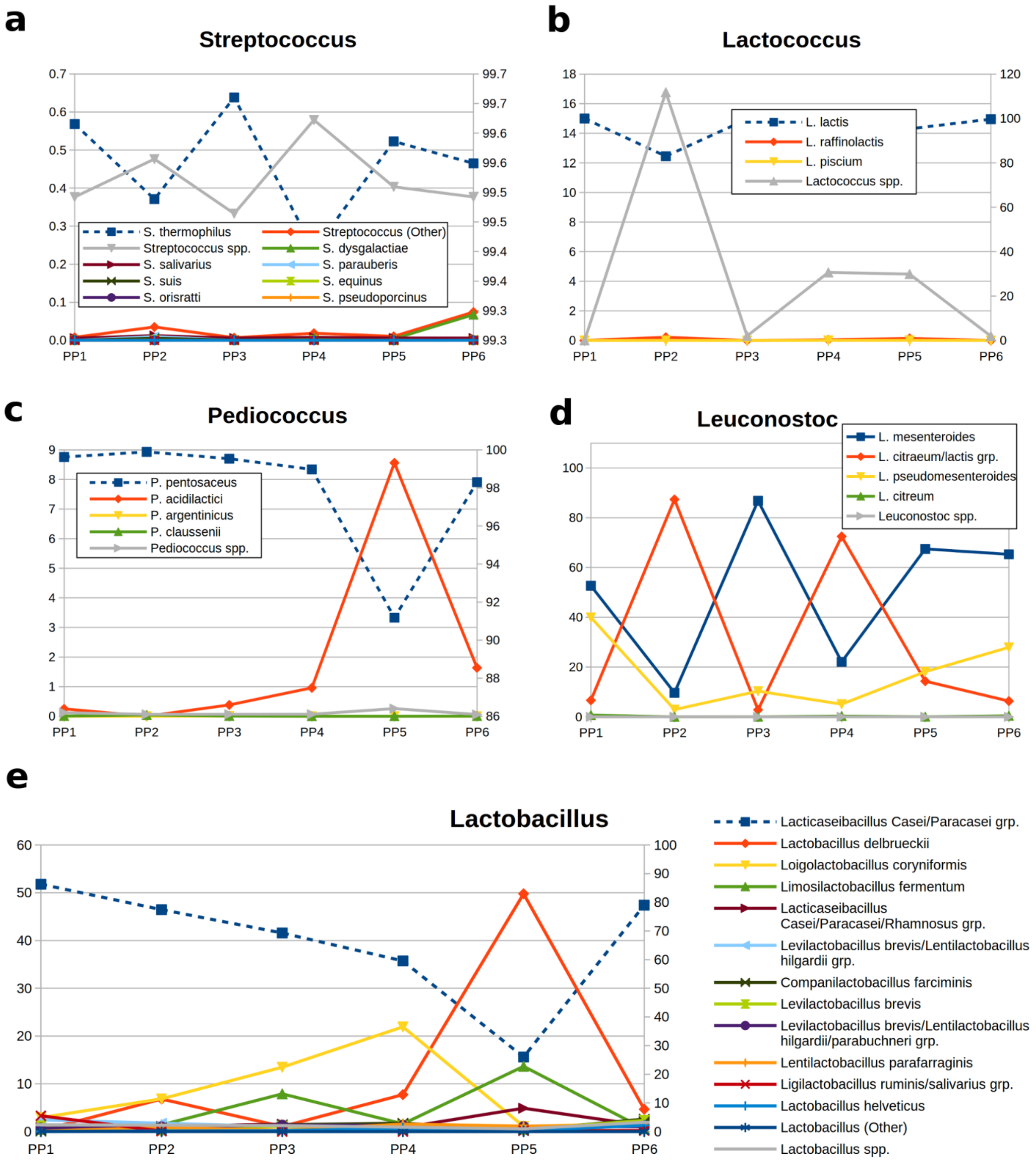

Supplementary Figure 3. Species-level characterization plots for five selected genera: (**a**) *Streptococcus*, (**b**) *Lactococcus*, (**c**) *Pediococcus*, (**d**) *Leuconostoc* and (**e**) *Lactobacillus*. The average proportion (Y-axis, n=9) of the most significant species per each genus along the six Pasture area-Producer (X-axis) is reported. Minor components of each genus are represented in "Other sp." category. Where necessary, due to a different scale, data were represented on secondary Y-axes (dashed lines).
