# Supplementary material for: High biodiversity in a limited mountain area revealed in the traditional production of Historic Rebel cheese by an integrated microbiota–lipidomic approach
Source: Sci Rep. 2021 May 14;11:10374. doi: 10.1038/s41598-021-89959-x (PMC8121794; doi:10.1038/s41598-021-89959-x)
Supplement: Supplementary file 3 — Supplementary Information. [file 41598_2021_89959_MOESM3_ESM.docx]

**Supplementary Methods**

**Species-level analysis**

Classification of reads belonging to the main bacterial genera of the collected samples was further improved, where possible, down to the species level, via a BLAST-based re-classification on an *ad-hoc* built reference database, based on the sequences available from NCBI RefSeq database for bacteria (<ftp://ftp.ncbi.nlm.nih.gov/genomes/refseq/bacteria/>), which comprised, as of 2019, December 15th a total of 27,334 species, belonging to 2,926 genera. These five genera (i.e.: Streptococcus, Lactobacillus, Lactococcus, Pediococcus, Leuconostoc) accounted for more than 90% of total relative abundance on average. As of April 2020, genus Lactobacillus underwent major re-classification into 25 different genera, including 23 novel ([**S1**]). Taxonomy classification in this paper was performed according to the previous classification due to how 16S rRNA reference databases were built. In species-level characterization, however, species have been re-named, where needed, according to the new nomenclature thanks to a specific web-based tool (http://lactotax.embl.de/wuyts/lactotax/)

*Reference sequences*

Through a custom script, for each genus, sequenced genomes for all species and strains were downloaded and properly formatted for further processing. For Lactobacillus, a careful review of the current literature has been performed in order to isolate, among all species, only those found associated to milk or cheese at least once. The following table reports which species have been included in the database:

| **New name** | **Old name** |  | **New name** | **Old name** |
| --- | --- | --- | --- | --- |
| Lactobacillus acidophilus | L. acidophilus |  | Lactobacillus jensenii | L. jensenii |
| Ligilactobacillus animalis | L. animalis |  | Lactobacillus johnsonii | L. johnsonii |
| Limosilactobacillus antri | L. antri |  | Lactobacillus kefiranofaciens | L. kefiranofaciens |
| Levilactobacillus brevis | L. brevis |  | Levilactobacillus parabrevis | L. parabrevis |
| Lacticaseibacillus casei | L. casei |  | Lentilactobacillus parabuchneri | L. parabuchneri |
| Loigolactobacillus coryniformis | L. coryniformis |  | Lacticaseibacillus paracasei | L. paracasei |
| Lactobacillus crispatus | L. crispatus |  | Lentilactobacillus parafarraginis | L. parafarraginis |
| Latilactobacillus curvatus | L. curvatus |  | Lactiplantibacillus plantarum | L. plantarum |
| Lactobacillus delbrueckii | L. delbrueckii |  | Limosilactobacillus reuteri | L. reuteri |
| Companilactobacillus farciminis | L. farciminis |  | Lacticaseibacillus rhamnosus | L. rhamnosus |
| Lentilactobacillus farraginis | L. farraginis |  | Ligilactobacillus ruminis | L. ruminis |
| Limosilactobacillus fermentum | L. fermentum |  | Latilactobacillus sakei | L. sakei |
| Fructilactobacillus fructivorans | L. fructivorans |  | Ligilactobacillus salivarius | L. salivarius |
| Lactobacillus gasseri | L. gasseri |  | Lactobacillus taiwanensis | L. taiwanensis |
| Schleiferilactobacillus harbinensis | L. harbinensis |  | Lactobacillus ultunensis | L. ultunensis |
| Lactobacillus helveticus | L. helveticus |  | Limosilactobacillus vaginalis | L. vaginalis |
| Lentilactobacillus hilgardii | L. hilgardii |  |  |  |

In all our analyses, only bacterial strains with a genome finishing grade of “Complete”, “Chromosome” or “Scaffolds” were considered. The following table summarizes the references used for each of the groups considered.

| **Genus name** | **Number of species** | **Number of strains** |
| --- | --- | --- |
| *Streptococcus* | 178 | 6,610 |
| *Lactobacillus* | 33 | 323 |
| *Lactococcus* | 6 | 30 |
| *Pediococcus* | 6 | 22 |
| *Leuconostoc* | 9 | 24 |

*Reads to re-classify*

From the zOTU table comprising all the samples, zOTUs classified within the above genera were selected and the sequences of all the reads grouped in each zOTU (were retrieved. In order to reduce the number of sequences to re-classify, clonal reads (i.e.: reads being identical throughout 100% of their length and composition) were grouped together, with the exception of reads belonging to Streptococcus genus, which were grouped at 99% similarity due to computational reasons.

*Classification*

Re-classification of the reads was performed through nucleotide BLAST (legacy BLAST, v 2.26) [**S2**], using a cutoff of 1e-10 for the e-value and de-activating the dust-filter. Only reads matching for at least of 80% of their length were retained and, for each read, the best match (i.e.: that or those with the higher bit-score) was selected.

In order to provide the best possible classification down to species-level, we tried to further resolve equivocal matches looking at which species they came from. Then, we created “clusters”, grouping together references showing identical scores on multiple reads. In this way, we were able to better discriminate Lactobacillus and Leuconostoc genera. Unclassified reads for Lactobacillus dropped from 54.6% to 1.2% and unclassified Leuconostoc dropped from 45.1% to <0.1%: the majority of previously unidentified Lactobacillus reads belonged to *Lacticaseibacillus casei/paracasei* group, whereas, for Leuconostoc, the majority fell into *Leuconostoc citraeum/lactis* group.

If a read still had multiple classifications on different species, the classification was reset to genus level.

*Relative abundance analysis*

In order to keep only consistent data for species-level evaluations, only samples having an incidence (i.e.: a relative abundance) higher than 0.5%, were considered. This was made to exclude samples with very few reads classified in the genus that could profoundly alter the dataset (e.g.: considering a sample in which we had only 1 read in a genus, this would have brought a 100% to the species-level classification for that certain species). Since the least sequenced sample had about 89,000 reads, this equaled having at least 178 reads in the genus. The following table summarizes the number of samples per each group that were kept:

| **Taxa** | **PP1** | **PP2** | **PP3** | **PP4** | **PP5** | **PP6** |
| --- | --- | --- | --- | --- | --- | --- |
| *Streptococcus* | 9 | 9 | 9 | 9 | 9 | 9 |
| *Lactobacillus* | 9 | 9 | 9 | 9 | 9 | 9 |
| *Lactococcus* | 1 | 8 | 3 | 7 | 7 | 3 |
| *Pediococcus* | 8 | 2 | 9 | 5 | 5 | 9 |
| *Leuconostoc* | 6 | 8 | 9 | 9 | 6 | 8 |

**Supplementary References**

[S1] Zheng J, Wittouck S, Salvetti E, Franz CMAP, Harris HMB, Mattarelli P, O'Toole PW, Pot B, Vandamme P, Walter J, Watanabe K, Wuyts S, Felis GE, Gänzle MG, Lebeer S. A taxonomic note on the genus Lactobacillus: Description of 23 novel genera, emended description of the genus Lactobacillus Beijerinck 1901, and union of Lactobacillaceae and Leuconostocaceae. Int J Syst Evol Microbiol. 2020 Apr;70(4):2782-2858. doi: 10.1099/ijsem.0.004107. Epub 2020 Apr 15. PMID: 32293557.

[S2] Altschul SF, Gish W, Miller W, Myers EW, Lipman DJ. Basic local alignment search tool. J Mol Biol. 1990 Oct 5;215(3):403-10. PubMed PMID: 2231712.
